# Oncocytoma of the Submandibular Gland: Diagnosis and Treatment Based on Clinicopathology

**DOI:** 10.1155/2016/8719030

**Published:** 2016-09-19

**Authors:** Betty Chen, Joshua I. Hentzelman, Ronald J. Walker, Jin-Ping Lai

**Affiliations:** ^1^Department of Otolaryngology-Head and Neck Surgery, Saint Louis University School of Medicine, St. Louis, MO 63104, USA; ^2^Department of Pathology, Saint Louis University School of Medicine, St. Louis, MO 63104, USA

## Abstract

*Background*. Submandibular oncocytomas are rare benign salivary gland neoplasms. They are typically found in Caucasian patients aged 50–70 years with no gender preference. Due to the overlapping histological and clinical features of head and neck tumors, they are often misdiagnosed.* Methods*. We report a case of unilateral submandibular gland oncocytoma in a 63-year-old Caucasian man.* Results*. The patient underwent unilateral submandibular gland resection and histopathologic analysis of the tumor specimen. On follow-up at 2 weeks and 1 year, no recurrence was identified.* Conclusion*. Submandibular oncocytomas are best diagnosed with preoperative FNA and CT imaging and have distinctive findings on cytology and histology. CT followed by fine-needle aspiration cytology would be the preferred diagnostic modalities. Due to its low rate of malignant transformation and recurrence, the best treatment is local resection with follow-up as necessary.

## 1. Introduction

Oncocytomas are rare benign neoplasms composed of oncocytes or polyhedral cells with eosinophilic cytoplasm made up of abundant mitochondria and dark centrally located nuclei [1–3]. Hürthle first described oncocytes in a canine thyroid gland in 1894 [4, 5]. The term “oncocytoma” was first used by Schaefer to describe “granular swollen cells” in ducts and acini of salivary glands [1, 6]. In 1931, Hamperl reported oncocytomas in numerous glandular structures including major salivary glands, thyroid and parathyroid glands, pituitary glands, testicles, pancreas, liver, and stomach [1, 7].

Salivary gland oncocytomas are primarily found in the parotid gland and rarely found in the submandibular glands [3]. To the best of our knowledge, there have only been 33 cases of submandibular oncocytoma reported in previous literature, including our case. Despite its rarity, submandibular oncocytoma is an important area of study because it has a distinct clinical course compared to more common salivary neoplasms such as pleomorphic adenoma and Warthin's tumor. Pleomorphic adenomas have 1.5% and 9.5% malignant potential on follow-up at 5 and 15 years, respectively [8], and can recur after resection [9]. In addition, 37 cases of carcinoma arising from previous Warthin's tumor have been reported [10]. In contrast, oncocytomas have extremely low malignant potential, and those in the submandibular gland have not been found to recur after surgery [11]. In other words, submandibular oncocytomas favor a better prognosis.

Submandibular oncocytomas can present asymptomatically or as tender, enlarging neck masses over weeks to years. Typical patients are Caucasians 50–70 years of age with no gender preference. There are no clear etiologies for the development of submandibular oncocytomas, although there have been cases associated with radiation exposure [11].

This report aims to evaluate the clinical and histopathological features of submandibular oncocytomas through a single case report at St. Louis University hospital and will include a review of previous literature with an emphasis on diagnostic criteria and future treatment of such cases.

## 2. Case Presentation

**Figure 1:**
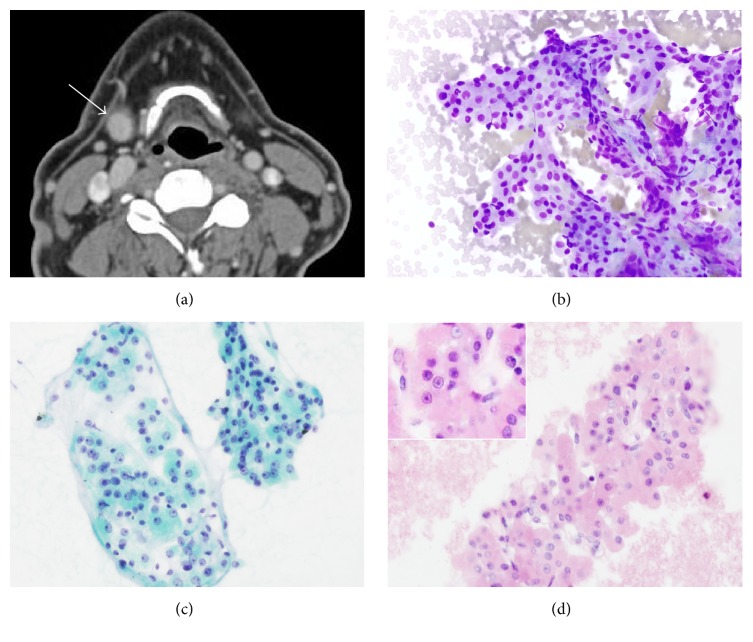
Imaging and cytopathology of the submandibular oncocytoma. (a) CT scan showing a well-circumscribed mass (1.6 × 1.3 cm) at the right submandibular space; (b)–(d) FNA of the mass showing clusters of polygonal eosinophilic epithelial cells with low N/C ratio, round nuclei, and prominent nucleoli ((b) Diff-Quik, ×400; (c) pap smear, ×400; and (d) cell block, ×400 (inset, ×600)).

A 63-year-old Caucasian male presented with a 3-year history of tender right neck mass. He denied other symptoms and his past medical history was noncontributory. He denied cigarette smoking and tobacco use and reported 15 alcoholic drinks per week. Past surgical surgery included an osteotomy of the clavicle. On physical exam, a 1.5 cm solid nodule was palpated in the right submandibular region above the tip of the hyoid. The presence of the mass was confirmed on CT imaging, which showed a well-defined, homogeneously enhancing 1.6 × 1.3 cm mass in the inferior pole of the submandibular salivary gland (Figure 1(a)).

A fine-needle aspiration (FNA) of the lesion was performed. In cytopathology (Figures 1(b)–1(d)), there were clusters of monotonous, polygonal, eosinophilic (oncocytic) epithelial cells with a low nuclear to cytoplasmic (N/C) ratio. The tumor cells had round nuclei and prominent nucleoli. There was no significant lymphoid population identified, which is commonly seen in Warthin's tumor. No mitotic figures or tumor necrosis were identified. Cytologic features were suggestive of submandibular oncocytoma.

**Figure 2:**
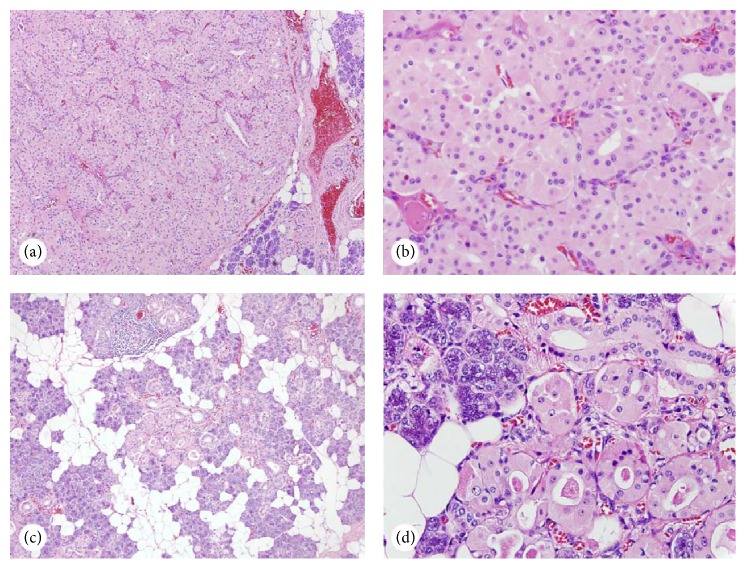
Histology of the submandibular oncocytoma. (a-b) The tumor is well circumscribed with a thin capsule ((a) ×100) and is composed of benign appearing oncocytes ((b) ×400); (c)-(d) foci of oncocytes present at the tumor adjacent submandibular tissue ((c) ×100; (d) ×400).

For definitive treatment and pathologic diagnosis, a right submandibular gland resection was performed. Gross examination revealed a weeping tan/yellow mass. The cut surface was coarsely lobulated with focal hemorrhage. Microscopically, the tumor showed a well-circumscribed mass with a thin capsule (Figure 2(a)). The tumor was composed of monotonous epithelial cells with a low N/C ratio, abundant eosinophilic cytoplasm, and round nuclei with prominent nucleoli (Figure 2(b)). Away from the mass within adjacent submandibular gland tissue were foci of oncocytic hyperplasia (Figures 2(c) and 2(d)). The patient was discharged on the same day following surgery. On the two-week follow-up visit, the patient reported no issues with the wound. On the one-year follow-up, no recurrence was identified.

## 3. Discussion

Oncocytomas of the salivary gland are rare benign neoplasms that comprise 3-4% of head and neck tumors [5, 20]. The majority of salivary gland tumors arise in the parotid gland (70%), followed by minor salivary glands (22%) and submandibular glands (8%) [5]. Submandibular oncocytoma is a very rare benign tumor that arises primarily in older Caucasian individuals aged 50–70 years. However, there have been cases reported in younger individuals, including a case involving a 19-year-old female [17]. According to previous cases of submandibular oncocytoma listed in Table 1, there is no gender preference, with a male-to-female ratio of approximately 1 : 1. In addition, the average age of diagnosis is comparable for both sexes, with males diagnosed at 59 years and females at 61 years. Submandibular oncocytoma most frequently presents as a painless enlarging mass, which was found in 48% (16/33) of cases, whereas 27% (9/33) involved a tender mass, and the rest had no data on symptoms.

Oncocytosis, marked by increased number of mitochondria, is frequently reported in aged, reactive, inflamed, hyperplastic salivary glands [21]. However, due to its rare incidence in submandibular glands, the etiology of submandibular oncocytomas remains unknown. One theory implicated the role of radiation in the pathogenesis of oncocytomas. In a follow-up study by Brandwein and Huvos, 20% (9/44) of patients with oncocytomas had radiation therapy or prolonged radiation exposure [11]. However, no conclusive evidence exists for the correlation between amount of radiation exposure and development of oncocytomas. Although rare in salivary glands, oncocytomas can be found mainly in the excretory ducts, also known as intercalated ducts, of minor salivary glands and parotid glands. Oncocytomas in the parotid glands may be derived primarily from reserve cells in intercalated ducts [22]. This is supported by immunohistochemistry data, which demonstrated the presence of CK7, CK8, and CK19, which are markers for human duct cells [22]. Submandibular gland oncocytosis may have a similar etiology, although research has mainly been focused on parotid gland oncocytomas.

The differential diagnosis for benign submandibular tumors includes pleomorphic adenoma and Warthin's tumor. Each tumor can be distinguished based on its histopathological characteristics. Oncocytomas are characterized by the presence of monomorphic oncocytes without mitoses and necrosis [11]. Unlike pleomorphic adenomas, which have thick and irregularly marginated capsules, oncocytomas have thin capsules, as seen in our case. Warthin's tumor can also be ruled out on cytology and histology by the lack of lymphatic population [12]. In addition to the primary tumor, surrounding areas of oncocytic metaplasia can be found [3]. This was seen in our patient, who had areas of oncocytic hyperplasia in the adjacent submandibular gland tissue. Submandibular gland oncocytomas have rare malignant potential. In 33 cases to date, only one reported malignant differentiation from a benign lesion [23]. Characteristics of malignant transformation include local invasion into muscular, perineural, and lymphatic structures as well as microscopic features including nuclear atypia, cellular polymorphism, mitoses, and focal necrosis [5].

Due to the similarities in clinical presentation between benign and malignant submandibular oncocytomas, radiologic imaging and fine-needle aspiration cytology (FNAC) are essential in distinguishing between the two entities. Ultrasound is recommended for initial assessment of a mass, but is insufficient because it does not provide information about surrounding structures. Recently, F-18 FDG PET/CT has shown promise in detecting features of salivary gland malignancies. Subramanian and colleagues described the utility of PET/CT in the initial staging and histologic grading of salivary gland malignancies [18]. Despite the superior spatial resolution and functional and anatomic data, there are limitations in using this modality. For instance, due to the lower maximum SUV in salivary glands, the detection accuracy of malignancies with lower F-18 FDG may be variable [18]. In addition, PET/CT is generally not indicated unless initial biopsy is concerning for malignancy. To date, neck CT with contrast is the preferred modality for evaluating the extent of invasion and spread of salivary gland tumors [20]. Fine-needle aspiration (FNA) is a common initial diagnostic procedure for investigating salivary gland masses due to its cost-effectiveness, simple technique, and fast results. FNA cytologic features of oncocytomas include uniformly polygonal, cytoplasm-rich cells with characteristic morphological features such as eosinophilic and granulated cells with round centralized nuclei [12]. Generally, no mitotic figures are identified on the cellblock in case other entities cannot be excluded. In addition, a cytology exam of the aspirate can be performed using immunohistochemistry. Benign and malignant tumors have been shown to have different activity of markers such as Ki-67, a nuclear protein expressed in proliferating cells indicative of active mitosis [5].

To date, the first-line treatment for submandibular oncocytomas is surgical excision. Of the cases in Table 1, all known treatments involved surgical resection, including unilateral or bilateral excision and radical resection, with no reported recurrence. Since areas of oncocytic hyperplasia may also be present in the tissue of the adjacent salivary gland, as in this case, resection of the whole gland is recommended. Submandibular oncocytomas have an extremely low potential of malignant transformation, with only one reported case. In addition, no local recurrences have been reported following resection [3, 11, 12, 20, 24]. Thus, radical dissection or adjuvant radiation therapy would not be necessary. Due to the rare incidence of these tumors, alternative methods of treatments such as medical managements have not yet been reported.

In summary, we present a case of submandibular oncocytoma, which is a rare benign salivary gland neoplasm. Distinguishing features of oncocytomas are best seen on preoperative FNA cytology and histology, which include the presence of monotonous oncocytes with low N/C ratio and lack of mitoses and necrosis. The malignant potential of a benign oncocytoma is extremely low at around 3%, with only one previously reported case in literature. CT followed by fine-needle aspiration cytology would be the preferred diagnostic modalities. Treatment is local excision of the tumor with appropriate follow-up as needed.

## Figures and Tables

**Table 1:** Summary of clinical characteristics of submandibular oncocytoma.

Case	Age (sex)	Signs/symptoms	Laterality	Size	Mode of diagnosis	Treatment	Follow-up
(1) Eneroth [12]	75 (F)	N/A	N/A	N/A	Aspiration biopsy	N/A	N/A
(2) Dibble and Sanford [13]	79 (M)	Asymptomatic, viral URI	Left	2 × 3 cm, grew to 5.5 × 3 × 2.5 cm	N/A	Excision via external method	N/A
(3) Mukai et al. [14]	61 (M)	N/A	Left	N/A	N/A	N/A	3 years, alive
(4) Goode and Corio [15]	60 (F)	N/A	Unknown	N/A	N/A	N/A	
(5) Brandwein and Huvos [11]	62 (M)	N/A	Left	N/A	N/A	N/A	6 months, alive
(6) Ziegler et al. [16]	56 (F)	N/A	N/A	N/A	N/A	N/A	9 months, alive
(7) Thompson et al.^*∗*^ 22 cases [3]	See descriptions below						
(8) Nakada et al. [2]	68 (M)	Painless, enlarging mass	Left	7 × 4.5 cm	FNA	Radical resection	1.5 years, alive
(9) Sakthikumar et al. [17]	19 (F)	Painless to dull ache	Left	3 × 5 cm	FNA	Excision	8 weeks, comfortable
(10) Subramaniam et al. [18]	85 (M)	Asymptomatic	Left	12 mm	F18 FDG PET/CT	N/A	N/A
(11) Dastaran and Chandu [19]	61 (F)	MEN2B, NF1 Long-standing mild tenderness	Bilateral	N/A	Ultrasound, FNA	Bilateral excision	1 year, no recurrence
(12) Chen et al. (present case)	63 (M)	Tender mass	Right	1.6 × 1.3 cm	FNA, CT	Excision	1 year, no recurrence

^*∗*^Thompson et al. [3] presented 22 cases of submandibular oncocytoma with 50 : 50 female-to-male ratio and an average age of 59 years. Sizes of the tumor ranged from 0.7 cm to 7 cm, averaging 3 cm. More than half of the cases (13/22) involved enlarging asymptomatic painless masses whereas the rest involved tender masses. On follow-up, none of the cases had evidence of recurrent disease.
